# The relationship between serum uric acid levels and liver stiffness in patients with type II diabetes mellitus and fatty liver disease

**DOI:** 10.1590/1806-9282.20241013

**Published:** 2024-12-16

**Authors:** Aysun Yakut

**Affiliations:** 1 Istanbul Medipol University, Sefakoy Hospital, Health Application and Research Center, Department of Gastroenterology – İstanbul, Türkiye.

**Keywords:** Uric acid, Type II diabetes mellitus, MAFLD, AST to platelet ratio index (APRI) score

## Abstract

**OBJECTIVE:**

High serum uric acid levels are associated with metabolic syndrome and diabetes mellitus. Several observational studies have shown the association between metabolic dysfunction-associated fatty liver disease and high serum uric acid. However, this association is controversial due to reverse causality. We aimed to investigate the relationship between the serum uric acid level and "aspartate aminotransferase-platelet ratio index score," which noninvasively shows the possible changes of metabolic dysfunction-associated fatty liver disease in the liver in patients diagnosed with type II diabetes mellitus.

**METHODS:**

This retrospective study was conducted with a total of 94 patients, 36 females and 58 males, who were hospitalized in the gastroenterohepatology outpatient clinic and diagnosed with hepatosteatosis and type II diabetes mellitus between January 2023 and January 2024. Laboratory tests, height, weight, body mass index, presence of fatty liver disease on ultrasound, and aspartate aminotransferase-platelet ratio index scores of the patients were examined.

**RESULTS:**

The mean serum uric acid level of the patients was 5.26±1.52 mg/dL, and the mean aspartate aminotransferase-platelet ratio index score was 0.26±0.13. The serum uric acid level was found to be associated with the hemoglobin A1c value (p=0.001; p<0.01). However, the aspartate aminotransferase-platelet ratio index scores of the patients did not show a statistically significant difference according to serum uric acid levels (p>0.05).

**CONCLUSION:**

No significant association was observed between serum uric acid and the noninvasive liver test aspartate aminotransferase-platelet ratio index score. Although a causal relationship between metabolic dysfunction-associated fatty liver disease and serum uric acid has been demonstrated in several studies, further research is needed to evaluate possible mechanisms in the liver.

## INTRODUCTION

Metabolic dysfunction-associated fatty liver disease (MAFLD) is a chronic liver disease characterized by the accumulation of fat in hepatocytes without alcohol consumption^1^. MAFLD is part of the pathological spectrum that progresses to simple fatty liver as well as cirrhosis and hepatocellular carcinoma^2^. MAFLD has shown a significant and progressive increase in the last 20 years^3^. Studies have shown that MAFLD, formerly known as nonalcoholic fatty liver disease, is associated with obesity, hyperlipidemia (HL), insulin resistance, type II diabetes mellitus (DM), and cardiovascular disease^4,5^.

Serum uric acid (SUA) is the end product of purine metabolism^6^. Many studies have shown a significant and strong association between metabolic syndrome (MetS), cardiovascular disease, type II DM, and chronic renal failure^7,8^.

Animal^9,10^ and observational^11–17^ studies have shown an association between high SUA levels and the risk of MAFLD. In a study conducted in the West, high SUA levels were found to be highly associated with MAFLD^17^. Similar results were shown in another study conducted in East Asia^16^. Moreover, some meta-analyses suggested that high SUA levels double the risk of MAFLD^18,19^. On the other hand, a cross-sectional study conducted in children and adolescents in South America found a correlation between SUA levels and MetS, but no correlation with MAFLD^20^. Another prospective study similarly found no association between SUA levels and MAFLD^21^. Based on these data, it is quite difficult to determine whether there is a causal relationship between SUA levels and MAFLD. In our study, we aimed to evaluate the relationship between SUA levels and APRI scores, which indicate the possible effects of MAFLD on the liver in patients with type II DM.

## METHODS

### Study design and participants

A retrospective observational study was conducted with 94 patients who applied to the gastroenterohepatology clinic between January 2023 and January 2024 and were diagnosed with type II DM and fatty liver disease on ultrasonography. Patients diagnosed with type II DM and confirmed with fatty liver disease on ultrasonography were included in the study. Patients who consumed alcohol, did not have fatty liver disease, were not diagnosed with type II DM, and were diagnosed with type I DM were excluded. Demographic characteristics of the patients (height, weight, body mass index, disease diagnoses, antidiabetic drugs, and other drugs used) were recorded from the hospital information system. Alanine aminotransferase (ALT), aspartate aminotransferase (AST), alkaline phosphatase, gamma-glutamyl transferase (GGT), hemogram, glucose, SUA, total cholesterol, high-density lipoprotein, low-density lipoprotein, and triglyceride (TG) values evaluated in the controls of patients diagnosed with type II DM were obtained from the hospital information system and recorded. SUA levels were considered normal in the range of 3.4–7.0 mg/dL for men and 2.4–6.0 mg/dL for women. Levels above 7.0 mg/dL in men and 6.0 mg/dL in women were accepted as high SUA. In addition, the APRI score, an indicator of noninvasive liver fibrosis, was calculated from these laboratory values. Body mass index (BMI) was calculated based on the height and weight of the patients. The study was designed according to the Strengthening Reporting of Observational Studies in Epidemiology (STROBE) guideline. Since the study concept was retrospective, it was conducted from the hospital database with the permission and approval of the hospital, and no additional consent was obtained from the patients. No questionnaire was applied to the patients.

Formulas used in the study

AST\ALT ratio

APRI score=[(AST/upper limit of normal AST range)X100]/platelet count

$\text{Body mass index}(\text{BMI})=\text{body weight}(\text{kg})/\text{height squared}({\text{m}}^{2})$

### Statistical analysis

The NCSS (Number Cruncher Statistical System) 2007 (Kaysville, Utah, USA) program was used for statistical analysis. Descriptive statistical methods (mean, standard deviation, median, frequency, percentage, minimum, and maximum) were used when evaluating the study data. The suitability of quantitative data for normal distribution was tested using the Shapiro-Wilk test and graphical analysis. The independent groups’ t-test was used to compare normally distributed quantitative variables between two groups, and the Mann-Whitney U test was used to compare non-normally distributed quantitative variables between two groups. The Pearson chi-square test and Fisher's exact test were used to compare qualitative data. Statistical significance was accepted as p<0.05.

### Ethics statement

This retrospective study was approved by the Istanbul Medipol University Non-Interventional Clinical Research Ethics Committee (Date: March 28, 2024, Number: 366). Guidance Recommendations for Medical Practitioners in Biomedical Research Involving Human Subjects have been prepared taking into account the Declaration of Helsinki.

## RESULTS

This study included 38.3% (n=36) female and 61.7% (n=58) male patients who were followed up in the gastroenterology outpatient clinic with the diagnosis of fatty liver disease and type II DM between January 2023 and January 2024. This study was conducted with a total of 94 patients. The ages of the patients ranged from 27 to 82, and the mean age was 56.9±10.7 (Table 1).

**Table 1:** Distribution of descriptive characteristics in a cohort of patients diagnosed with metabolic dysfunction-associated fatty liver disease and type II diabetes mellitus.

		n (%)
Sex	Female	36 (38.3)
	Male	58 (61.7)
Age	Mean±SD	56.9±10.7
	Median (min–max)	56 (27–82)
Height (m)	Mean±SD	1.61±0.09
	Median (min–max)	1.6 (1.4–1.8)
Weight (kg)	Mean±SD	81.49±15.50
	Median (min–max)	80 (48–130)
Body mass index (BMI) (kg/m^2^)	Mean±SD	31.37±6.11
	Median (min–max)	30 (19.6–52.4)
Waist circumference (cm)	Mean±SD	106.00±13.65
	Median (min–max)	104.5 (48–145)
Presence of obesity	None	46 (48.9)
	Present	48 (50.1)

SD: standard deviation; BMI: body mass index.

100% of the patients included in the study had type II DM, 60.9% (n=56) had HL, 60.6% (n=57) had hypertension (HT), 4.3% (n=4) had chronic heart failure, 10.6% (n=10) had coronary artery disease, 1.1% (n=1) had ischemic stroke, 6.4% (n=6) had peripheral neuropathy, 5.3% (n=5) had retinopathy, 4.3% (n=4) had peripheral arterial disease, and 2.1% (n=2) had chronic renal failure. The drugs used by the patients as antidiabetic drugs were as follows: 84.9% (n=79) metformin, 8.6% (n=8) acarbose, 29% (n=27) sulfonylurea, 14% (n=13) gliptin, 39.1% (n=36) insulin, and 1.1% (n=1) GLP-1 analogs. The drugs used by the patients as antihypertensive drugs were as follows: 26.4% (n=24) angiotensin-converting enzyme inhibitors, 22% (n=20) angiotensin receptor blockers, 15.4% (n=14) calcium channel blockers, 18.7% (n=17) beta-blockers, 38.5% (n=35) thiazides, 1.1% (n=1) furosemide, and 1.1% (n=1) alpha-blockers. 52.7% (n=48) of the patients were using statins as anti-lipemic drugs.

SUA levels of the patients were normal in 68.75% and high in 23.25%. The mean SUA level of the patients included in the study was 5.26±1.52 mg\dL, the mean ALT value was 25.60±14.23 U\L, the mean AST value was 20.69±7.72 U\L, and the mean AST/ALT ratio was 0.90±0.30. The mean APRI score of the patients was found to be 0.26±0.13 (Table 2).

**Table 2:** Distribution of laboratory measurements in the patient cohort diagnosed with metabolic dysfunction-associated fatty liver disease and type II diabetes mellitus.

	Mean±SD	Median (min–max)
CRP (mg/L)	1.10±2.09	0.6 (0–14.8)
Albumin (gr/dL)	4.18±0.44	4.2 (2.2–5.1)
ALT (U/L)	25.60±14.23	22 (7–104)
AST (U/L)	20.69±7.72	19 (11–54)
AST/ALT	0.90±0.30	0.9 (0.3–2.3)
APRI	0.26±0.13	0.2 (0.1–0.7)
Total cholesterol	205.77±41.21	202.5 (123–297)
LDL (mg/dL)	121.91±32.23	117.5 (65–203)
HDL (mg/dL)	43.58±10.82	42 (23–77)
TG (mg/dL)	203.03±185.95	153 (43–1,160)
HbA1c (%)	7.84±2.29	7.2 (5.2–14)
Creatinine (mg/dL)	0.88±0.24	0.8 (0.6–2.1)
SUA (mg/dL)	5.26±1.52	5.1 (2.5–10.2)
PLT	273.02±75.68	263 (115–556)
SBP (mm/Hg)	131.16±14.78	130 (100–170)
DBP (mm/Hg)	76.32±12.30	73.5 (60–100)
MBP	94.60±11.98	95 (73.3–120)

CRP: C-reactive protein; ALT: alanine aminotransferase; AST: aspartate aminotransferase; AST/ALT: aspartate aminotransferase/alanine aminotransferase; APRI score: aspartate aminotransferase (AST)-platelet ratio index; LDL: low-density lipoprotein; HDL: high-density lipoprotein; TG: triglyceride; HbA1c: hemoglobin A1c; SUA: serum uric acid; PLT: platelet; SBP: systolic blood pressure; DBP: diastolic blood pressure; MBP: mean blood pressure; SD: standard deviation.

The rate of HT in patients with high SUA levels was found to be statistically significantly higher than in patients with normal SUA levels (p=0.013; p<0.05). The rate of thiazide use as an antihypertensive in patients with high SUA levels was found to be statistically significantly higher than in patients with normal SUA levels (p=0.011; p<0.05). Hemoglobin A1c (HbA1c) values of patients with high SUA levels were found to be statistically significantly higher than in patients with normal SUA levels (p=0.001; p<0.01) (Table 3).

**Table 3:** Evaluation of laboratory measurements according to uric acid values.

		Serum uric acid		p
		High (n=68)	Normal (n=23)	
APRI score	Mean±SD	0.27±0.14	0.24±0.09	c **0.394**
	Median (min–max)	0.2 (0.1–0.7)	0.2 (0.1–0.5)	
AST/ALT	Mean±SD	0.87±0.26	1.00±0.39	c **0.190**
	Median (min–max)	0.8 (0.3–1.4)	0.9 (0.5–2.3)	
MBP	Mean±SD	95.25±12.38	92.59±10.66	c **0.347**
	Median (min–max)	93.3 (73.3–120)	96.7 (76.7–111.7)	
HbA1c	Mean±SD	8.29±2.38	6.44±1.21	d **0.001** **
	Median (min–max)	7.5 (5.2–14)	6.3 (5.2–11.1)	

c Mann-Whitney U test.

d Student t-test.

** p<0.01.

APRI score: aspartate aminotransferase (AST)-platelet ratio index; AST/ALT: aspartate aminotransferase/alanine aminotransferase; MBP: mean blood pressure; HbA1c: hemoglobin A1c; SD: standard deviation. HbA1c of cases with high uric acid was higher than those with normal levels (p=0.001; p<0.01). APRI score, AST/ALT ratio, and MBP values of cases according to uric acid groups were not statistically significant (p>0.05).

## DISCUSSION

MAFLD is considered the hepatic variant of MetS. Obesity, HL, and insulin resistance are important factors in the etiology of MAFLD. Various studies have suggested that insulin resistance, de novo lipid synthesis, and the oxidant effect are potential pathways linking SUA levels to the formation of MAFLD. It has also been reported that SUA contributes to hepatic steatosis and insulin resistance by causing mitochondrial oxidative stress^22,23^. On the other hand, the study by Li et al.^24^ showed that MAFLD increases causally with SUA levels, but there is no causal relationship between SUA levels and MAFLD risk according to the Mendelian randomization method. This study investigating the causal relationship between SUA levels and MAFLD risk was conducted according to the two-way Mendelian randomization analysis. Although the conclusion drawn from this study showed that the presence of MAFLD may increase SUA levels, no evidence was shown regarding the risk of MAFLD due to high SUA levels^24^. Another study conducted in Denmark investigated whether SUA levels could be a biomarker for MAFLD in pediatric obese patients. In this study, SUA levels were found to be associated with TGs, fat-free mass, and GGT. However, no correlation was found between the SUA level and MAFLD^25^. The relationship between SUA levels and mortality was examined in MAFLD patients without severe renal failure in the USA. In this prospective study, no relationship was shown between SUA levels and survival in the 26.58-year follow-up of MAFLD patients^26^. The mean BMI of patients diagnosed with type II DM in our study was 31.37 kg/m^2^. The relationship between the presence of type II DM, HbA1c value, and SUA level was found to be similar to the literature (p=0.01, p>0.05). Notably, 70% of SUA clearance is provided by renal excretion^26^. Most of our patients did not have a diagnosis of chronic renal failure. Therefore, there was no renal pathology that could affect SUA clearance. The mean SUA level of our patients was 5.1 mg/dL (min=2.5–max=10.2). Ultrasonographically, the patients had fatty liver disease. The APRI score was 0.27±0.14 in the group with high SUA levels and 0.24±0.09 in the group with normal SUA levels. The APRI score in our MAFLD patient cohort was F0–F1. This study showed that there was no statistical difference between SUA levels and APRI scores in MAFLD patients (p=0.394, p>0.05). Although the controversial relationship between MAFLD and SUA levels has been shown in animal experiments, retrospective studies, meta-analyses, and randomized trials, there is no study showing the relationship between SUA levels and liver stiffness in MAFLD patients. The strength of our study is that it is the first study to show the relationship between SUA levels and liver stiffness in MAFLD patients.

The limiting factors in our study were the small size of our patient cohort, the fact that most of our patients had low SUA levels, and the fact that it was a retrospective study. On the other hand, the fact that a more objective noninvasive test such as fibroscan^®^, which shows liver damage in patients with MAFLD, is not available in our center and not in every center was the limiting factor in our study.

## CONCLUSION

There was no relationship between SUA levels and APRI scores, which evaluates liver stiffness, in patients diagnosed with type II DM and MAFLD.

## Data Availability

The database of the study can only be used upon the written and justified request of the relevant author.
